# Scaling-up problem management plus for refugees in Switzerland - a qualitative study

**DOI:** 10.1186/s12913-023-09491-8

**Published:** 2023-05-15

**Authors:** Julia Spaaij, Daniela C. Fuhr, Aemal Akhtar, Luisa Casanova, Tobias Klein, Matthis Schick, Sonja Weilenmann, Bayard Roberts, Naser Morina

**Affiliations:** 1 Department of Consultation-Liaison Psychiatry and Psychosomatic Medicine, University Hospital Zurich, University of Zurich, Culmannstrasse 8, Zurich, CH-8091 Switzerland; 2 Department of Health Services Research and Policy, London School of Hygiene and Tropical Medicine, London, UK; 3 Department of Prevention and Evaluation, Leibniz Institute for Prevention Research and Epidemiology, Bremen, Germany; 4 Health Sciences, University of Bremen, Bremen, Germany; 5 School of Psychology, University of New South Wales, Sydney, Australia; 6 Department of Clinical Neuroscience, Division of Insurance Medicine, Karolinska Institutet, Stockholm, Sweden

**Keywords:** Mental health, Scale-up, Refugees, Asylum seekers, Problem management plus, Lay-provider, Task-shifting

## Abstract

**Background:**

Refugees are at an increased risk of developing symptoms of mental disorders but face various structural and socio-cultural barriers to accessing mental health care. The SPIRIT project (Scaling-up Psychological Interventions in Refugees In SwiTzerland) seeks to promote the resilience of refugees and improve their access to mental health care. For this purpose, Problem Management Plus (PM+), an evidence-based low-intensity psychological intervention delivered by trained non-specialist “helpers”, is being scaled-up in Switzerland.

**Objective:**

To identify factors influencing the process of the large-scale implementation of PM + for refugees in Switzerland and to develop recommendations to guide the implementation process.

**Methods:**

22 semi-structured interviews were conducted with key informants (Syrian refugees who previously participated in PM+, PM + helpers, health professionals working with refugees and decision-makers from the migration, integration, social, and health sectors). The data were analyzed using thematic analysis, combining an inductive and deductive approach.

**Results:**

The data revealed three major themes, which might have an impact for the longer-term implementation of PM + in Switzerland. First, preconditions for successful integration in the health system prior to scaling-up such as sustainable funding or the introduction of a stepped care approach. Second, the requirements for the PM + intervention supporting scale-up such as quality control during PM + delivery, PM + modality, time and setting when PM + is offered or the views on task sharing. Third, the perceived benefits of scaling-up PM + in Switzerland.

**Conclusions:**

Our results have shown that PM + must be scaled-up within a stepped care approach, including a functioning triage system and sustainable funding. Rather than selecting one modality or setting, it seemed preferable to offer a variety of formats and settings to achieve maximum reach and benefits. A successful scale-up of PM + in Switzerland might have various benefits. Communicating them to policy-makers and health providers, might enhance their acceptability of the intervention and their willingness to adopt PM + in regulatory structure and promote it.

**Supplementary Information:**

The online version contains supplementary material available at 10.1186/s12913-023-09491-8.

## Introduction

The number of forcibly displaced persons worldwide has reached 100 million for the first time [1]. While most of these individuals have been internally displaced, approximately 30 million are refugees and asylum seekers who have crossed an international border [2]. Prior to and during their flight, refugees are frequently exposed to potentially traumatic events, such as experiences of war and persecution, death of loved ones or experiences of violence during flight [3, 4], associated with a high prevalence of mental disorders such as depression, anxiety, or posttraumatic stress disorder [5–7]. Once resettled in host countries, they experience post-migration living difficulties, such as discrimination, language problems, and separation from family [8–12]. These post-migration stressors may further exacerbate symptoms of mental disorders [5, 6, 13–15]. While the relationship between exposure to traumatic events and mental distress has long been established [16–20], more recent research focuses on the effect of post-migration stressors on the mental health of refugees and asylum seekers [3, 10, 21, 22].

There are currently around 130,000 refugees and asylum seekers in Switzerland [23], excluding approximately 70,000 Ukrainian refugees who have come to Switzerland after the start of the war in Ukraine in February 2022 [24]. Despite frequent symptoms of mental disorders, refugees in Switzerland have a low uptake of mental health services [25]. By means of mandatory health insurance, refugees are entitled to the same health care as the general population, however, they face structural barriers such as the lack of specialized treatment facilities and limited funding for interpreters, as well as several socio-cultural barriers to healthcare (e.g., mental health stigma ormental health illiteracy) [26]. These barriers might result in untreated mental disorders and thus, could lead to a clinical deterioration and chronification of symptoms. Research has shown that impaired mental health is associated with poor social and economic integration [9, 27, 28]. In consequence, chronic mental health conditions may also result in greater economic and social burdens for the host nation [29]. To mitigate these barriers to mental health care and enable local health systems to provide refugees facilitated access to mental health care, task sharing initiatives may be a complementary solution [30]. Task sharing initiatives delegate specific healthcare tasks from specialized staff (e.g., psychiatrists or psychotherapists) to healthcare workers with less extensive training or even to trained lay-people [31].

One example of a task sharing intervention is Problem Management Plus (PM+), a transdiagnostic low-intensity intervention developed by the World Health Organization (WHO) [32]. The intervention consists of five 90-minute sessions and is delivered by trained non-specialist “helpers” who share the same cultural background and language as the participants [32]. Over the five PM + sessions, participants are taught four strategies, (a) stress management, (b) problem management, (c) behavioral activation and (d) strengthening social support). The final session focuses on relapse prevention. PM + can be carried out in individual or group format [33, 34]. Although it has often been delivered face-to-face, it has recently been adapted to allow for remote delivery via videoconferencing tools [35, 36]. The intervention was initially developed for, and successfully evaluated in, low- and middle-income countries with limited mental health care resources [37–40]. More recently, as part of the STRENGTHS project which tested the intervention for Syrian refugees in various countries in Europe and the Middle East, the feasibility, effectiveness and cost-effectiveness of the PM + intervention has been investigated through trials with refugees in high-income countries [41–44].

The results of the STRENGTHS project have shown that PM + is an acceptable, feasible and safe treatment option for refugees and asylum seekers in high-income countries. Additionally, the intervention is effective in reducing psychological distress and enhances psychological functioning compared to participants in control conditions [41, 42, 45, 46]. Following these positive outcomes, the SPIRIT (Scaling-up Psychological Interventions in Refugees in Switzerland) project was launched.

SPIRIT seeks to improve access to mental health care for refugees in Switzerland by scaling-up the low-intensity intervention PM + in all six asylum regions in Switzerland. According to the WHO, the process of scaling-up refers to *“deliberate efforts to increase the impact of successfully tested health innovations so as to benefit more people and to foster policy and programme development on a lasting basis”* [47]. This means that successfully tested interventions, such as PM+, are implemented in “real world settings” to increase the reach of the intervention and to ensure long-term sustainability. Scaling-up mental health interventions has been proposed as one mean to reduce the mental health treatment gap [48, 49].

To explore the scalability of PM + and guide the process of scaling-up, information needs to be gathered not only on the effectiveness of PM + but also on the experiences and perceived impact of refugees who would be receiving these services. In addition, experiences of implementers are important to understand the translation of the delivery of PM + into existing mental health care systems, and policy makers who would be able to assist in achieving these goals. Thus, the present study aimed to identify factors influencing the Switzerland-wide scale-up of PM + for refugees and to give recommendations to guide the implementation process.

## Methods

### Setting

#### Recruitment and sample

Semi-structured interviews were conducted with four different groups of key informants (KI) in Switzerland. These KIs were (a) Syrian refugees who had previously participated in the PM + intervention in the STRENGTHS trial, (b) PM + helpers who had previously delivered PM + as part of the STRENGTHS project, (c) healthcare providers working with refugees and asylum seekers, and (d) policy makers from the migration, integration, social, and health sector. We aimed at interviewing approximately 20 KIs (i.e. five per group). Interviewees from groups *a* and *b* were selected from participants and PM + facilitators of a prior randomized controlled trial on PM + in Switzerland [44].

To allow for a wide variety of results, KIs were recruited following the principles of maximum variation sampling [50] with regard to age and place of work. In addition, we aimed for an equal gender ratio across all groups. Based on these principles and KIs’ knowledge on refugee mental health, experience with the PM + intervention or knowledge on mental health policies in Switzerland, they were selected through their participation in previous PM + research or through the professional network of the research team members. Interviewees needed to speak one of the study languages (German, English or Arabic) and had to be aged 18 years or older.

#### Study procedures

**Development of topic guides.** Interviewers (two master students and an Arabic speaking research assistant) used three topic guides for: (1) the PM + participants, (2) the PM + helpers and (3) the healthcare providers and policy makers, which included questions regarding the implementation of all PM+. The questions reflected our previously developed conceptual framework on the scaling-up of psychological interventions (based on [51–53]) and previous experience with the PM + intervention (e.g., experiences from the pilot RCT in Switzerland [41]). All topic guides were pilot tested before the data collection and adapted, if needed. The final versions of the topic guides are included in the appendix.

**Data collection.** The study was carried out at the Outpatient Clinic for Victims of Torture and War of the University Hospital Zurich. Interviews were conducted between June and August 2021. The interviews lasted around 60 min and were conducted individually. They were organized either face to face or remotely, based on the KI’s preference. Face-to-face interviews were conducted at the University Hospital Zurich or at one of the participants’ institutions. Remote interviews were held either via Skype for Business or via telephone. Informed consent was obtained orally prior to participation and recorded for the purpose of documentation. All interviews were audio-recorded. Interviewees were reimbursed with a voucher worth 40 CHF (approximately 40 USD).

The interviews for PM + participants and helpers were conducted in Arabic by Arabic-speaking research assistants. Interviews were conducted in German or English for healthcare providers and policy makers by LC and TK. The interviewers took field notes, if necessary. The recordings and the field notes were safely stored on the University Hospital Zurich servers.

#### Data analysis

Audio recordings were either transcribed verbatim or, in case of the interviews in Arabic, directly translated to English by a professional translator. Data were analyzed following thematic analysis, combining a deductive and a content-driven inductive approach [54]. First, researchers familiarized themselves with the data and identified codes. Some codes, themes and subthemes were derived deductively from questions in the topic guide (e.g., modality or time and setting where PM + is offered) while in the inductive approach, identified through meaningful patterns in the codes and that were combined into themes and associated subthemes and codes, highlighting specific elements of identified themes (e.g., codes regarding views on task sharing). The coding framework was piloted and subsequently revised.

Data analysis was performed by LC and TK using NVivo [55]. Two independent coders (LC and TK) each applied the final codebook on approximately 20% the data. The intercoder reliability was к = 0.89. The high intercoder reliability allowed to equally divide the remaining 80% of the data among the two coders (LC and TK). JS reviewed their coding and included final adaptions.

## Results

A total of *N* = 22 interviews were finally conducted with PM + participants (*n* = 5), PM + helpers (*n* = 5), mental health professionals (*n* = 5) working with refugees and policy makers (*n* = 5). Mean age of the interviewees was 37 years for group (a) (*M* = 36.6, *SD* = 11.14, range: 26–55), 39 years for group (b) (*M* = 39.4, *SD* = 7.60, range: 28–48), 44 years for group (c) (*M* = 43.8, *SD* = 6.98, range: 35–62) and 40 years for group (d) (*M* = 39.8, *SD* = 7.19, range: 33–52). 13 out of the 22 interviewees were female (59%). A table summarizing the characteristics of the interviewees can be found in Table 1.

**Table 1:** Interviewees’ characteristics

Interviewee	Gender	Professional background*	Region*,**
*Healthcare provider 1*	Female	Physician at federal asylum center	1
*Healthcare provider 2*	Female	Psychotherapist and medical director of an outpatient unit for refugees and asylum seekers	2
*Healthcare provider 3*	Female	Nurse at a federal asylum center	1
*Healthcare provider 4*	Female	Psychiatrist	2
*Healthcare provider 5*	Male	Psychiatrist	2
*Healthcare provider 6*	Female	Physician	1
*Healthcare provider 7*	Male	GP for refugees and asylum seekers	1
*Policy maker 1*	Female	Medical expert working in a governmental institution for migration issues	1
*Policy maker 2*	Male	Specialist working at the welfare department	1
*Policy maker 3*	Female	Medical specialist working at a cantonal department of health	1
*Policy maker 4*	Female	Medical specialist working at a cantonal department of health	1
*Policy maker 5*	Male	Division manager of a transit center for refugees	1
*Helper 1*	Male	-	1
*Helper 2*	Male	-	1
*Helper 3*	Female	-	1
*Helper 4*	Female	-	1
*Helper 5*	Female	-	1
*Participant 1*	Female	-	1
*Participant 2*	Male	-	1
*Participant 3*	Male	-	1
*Participant 4*	Female	-	1
*Participant 5*	Male	-	1

* Group c & d only

** Region (1 = German-speaking region, 2 = French-speaking region)

**Fig. 1:**
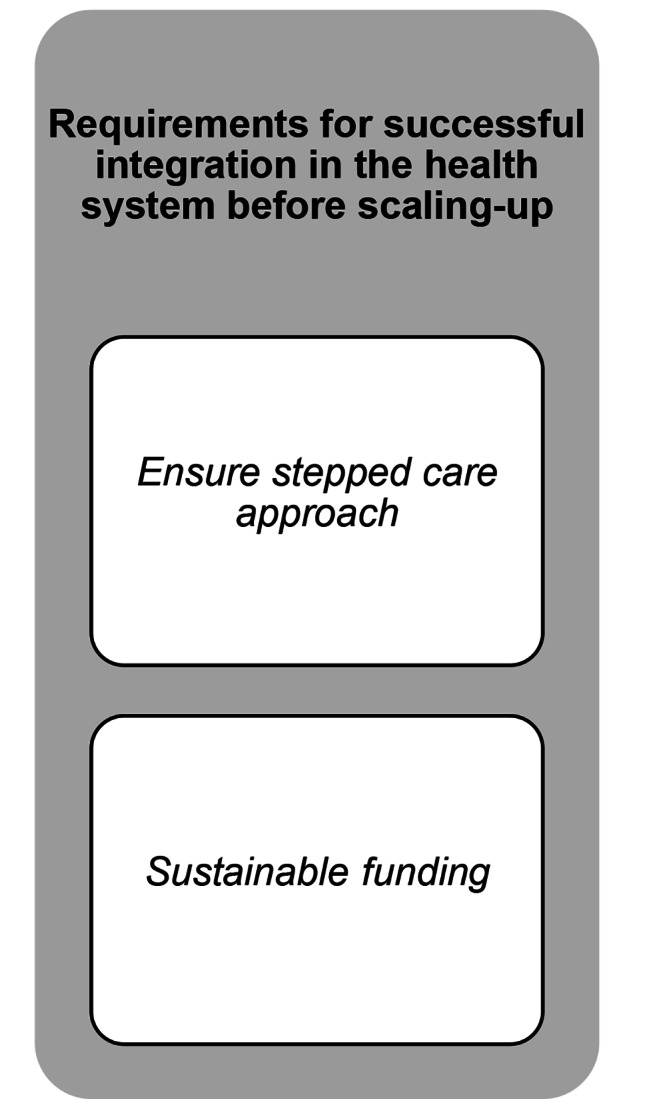
Overview of the first theme and the related subthemes.

**Fig. 2:**
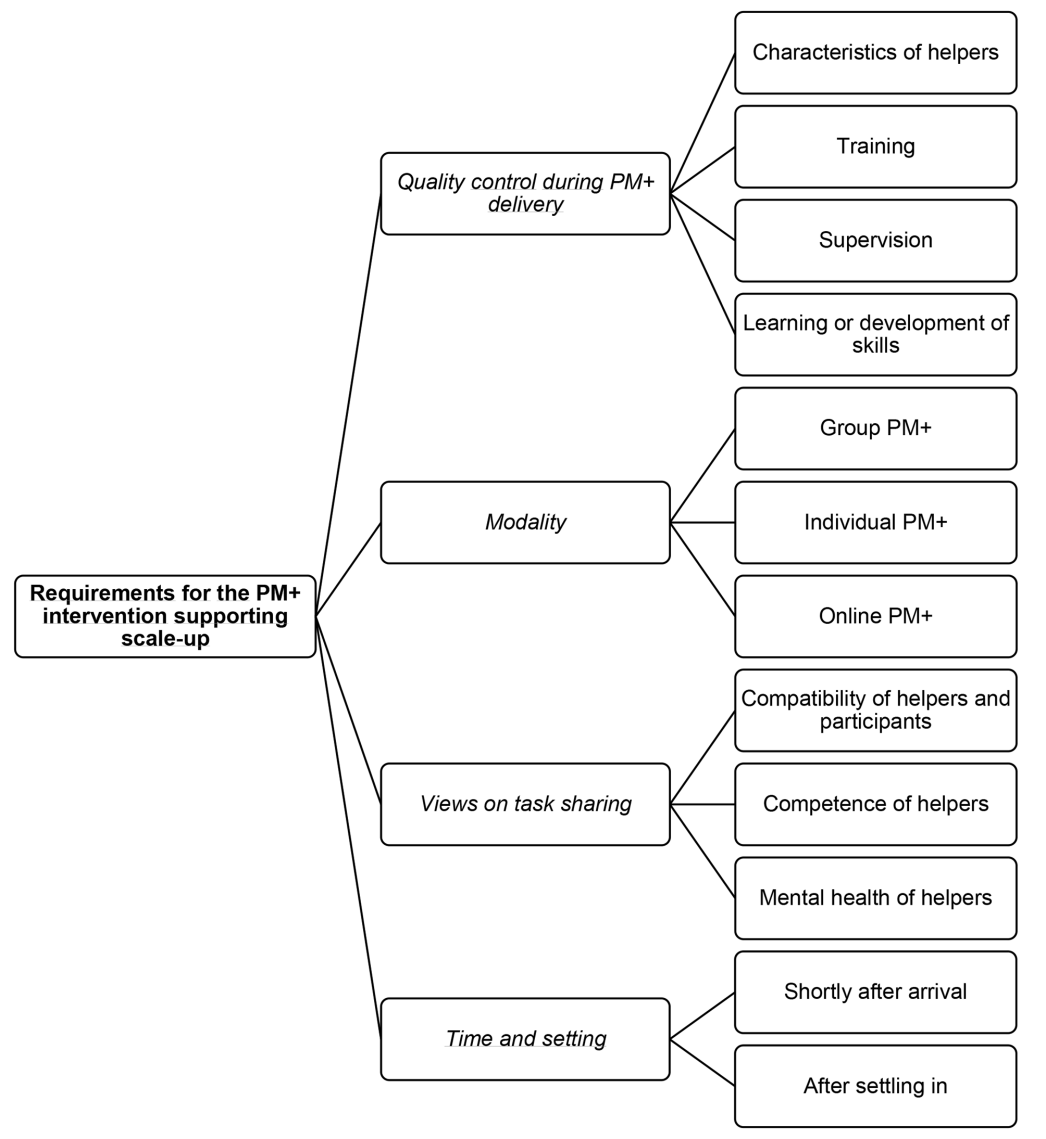
Overview of the second theme and related subthemes.

**Fig. 3:**
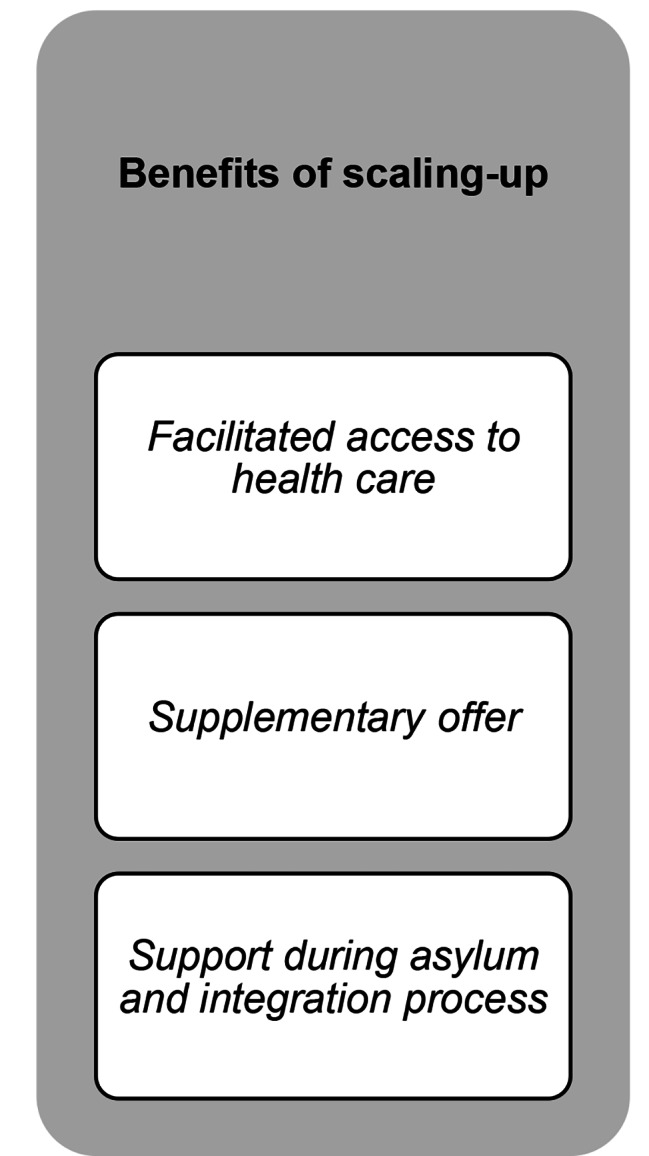
Overview of the third theme and related subthemes.

The results revealed three themes: (i) requirements for successful integration in the health system before scaling-up; (ii) requirements for the PM + intervention supporting scale-up; and (iii) benefits of scaling-up, each with related subthemes and codes. The final codebook including all themes, subthemes and codes with example quotations can be found in the appendix. An overview of the results can be found in Figs. 1 and 2, and Fig. 3.

## Theme 1: requirements for successful integration in the health care system before scaling-up

### Ensure a stepped care approach

One important requirement for successful integration in the health care system prior to scaling-up was to embed the intervention in already existing structures and ensure the PM + intervention is integrated in a stepped care approach with clearly defined steps.

A frequently mentioned point was the triage of individuals through initial assessment to identify who would benefit from low-intensity psychological interventions such as PM + and who may need more intensive treatment (e.g., trauma-focused psychotherapy). A healthcare provider (6) noted how “*something like PM + can be very good and can be sufficient. You just have to select well. I think in this concern, triage is the most important thing. For whom it is really a good fit and for whom it is not”*. One barrier identified that may hinder implementation is long waiting times that exist for mental health care in Switzerland. If an initial assessment indicates that someone is experiencing severe distress, or if they are still experiencing distress following the completion of PM+, other services need to be immediately available, as discussed by one healthcare provider (2):

*“If a low-intensity intervention reveals that cases are more severe, then it must also be possible to [offer further treatment]. […] It’s all very well to say after 5 sessions that this person actually needs psychotherapy. But what do you do next? […] So, you ask yourself, what’s the use of an early diagnosis if you can’t follow up on it? It’s like when you say you’re screening for cancer, and then you say: you do have cancer, but we can’t operate on it”.*

Another essential element was networking and collaborating with other mental health services and providers. While policymaker 4 said that she *“could imagine that the people who work in the system are more grateful if they also have something […] to refer to”*, she mentioned that one barrier to the implementation of PM + might be the competition with already existing treatment services. Similarly, policymaker 1 indicated that for successful implementation of the intervention, PM + would need to be demarcated from existing services. This would include *“demarcation to existing offers but also existing professional groups”*. To ensure good collaboration, it seems important to educate healthcare providers about PM + and to integrate healthcare providers and their needs into the implementation process.

### Sustainable funding

Interviewees mentioned that a large barrier hindering successful implementation of interventions such as PM + is sustainable funding. One policy maker (2) stated that *“the care and support of refugees is a state task. […] if [PM+] can be [integrated] within the regulatory structures, as in health insurance, then it is simply easier to launch and use such offers”*. Similarly, one healthcare provider (3) said : *“One barrier to implementation could be the costs. Does it cost anything? What does it cost? […] Is it covered or not covered by the health insurance? It’s all about money, really”.*

Funding through the private sector was seen as problematic, as it does not guarantee a sustainable implementation. A policy maker (2) noted how *“a private institution that drops out and then you would have to stop the program, that would be a shame. The commitment would have to be long-term. For me, it is clearly a government task”*. One policy maker (3) mentioned that a barrier for a sustainable implementation might be that *“PM + falls between responsibilities at the structural level”*. This was also reflected by others regarding the responsibility for the implementation of PM+. While some argued that PM + should therefore be covered by the health care insurances, policy maker 4 pointed out the “preventive character” of PM+, which contrasts the existing healthcare system in Switzerland, as Swiss health insurance does not usually cover preventive services: “*I would say that the health sector should be [less responsible], because the aim [of PM+] should be to have a preventive effect. That you can even keep people out of the health sector. Or if so, to stabilize them with low intensity offers”*.

## Theme 2: requirements for PM + intervention supporting scale-up

### Quality control during delivery of PM+

#### Characteristics of PM + helpers

The characteristics of the PM + helpers were identified as an important requirement for both scale-up and quality control during the delivery of the intervention. An important selection criterion identified by both PM + helpers and policy makers was sufficient knowledge of both German and the language of implementation. Healthcare provider 6 said that “*one of the most difficult things is to find someone […] who speaks the language really well. Who also understands it really well”*. One helper (2) acknowledged that a certain level of education would be necessary to follow this training: “*I suppose that uneducated people, those who haven’t been to a college or university, would face difficulties*”. Helper 4 identified additional criteria (e.g., social skills, openness, resilience) that helpers should possess to be successful: *“The selection criteria shouldn’t be limited to having a university diploma and having spent a few years as a refugee in Switzerland. There are additional criteria that need to be taken into consideration. […] Trustworthiness, for instance, as well as awareness and active listening skills, management skills, punctuality and readiness when submitting their paperwork, and so on. A certain foundation or basis”.*

Additionally, helper 2 suggested that to improve the selection process of new helpers, more experienced helpers should be present and actively take part in the selection process.

#### Training of helpers

Helpers mentioned several suggestions on how the improve the training of future helpers and to ensure that the quality of the intervention can be maintained long-term. Helper 2 stated that the implementers could “*help by providing [new helpers] with real-life situations/examples during their training. There are many cases […], which we can share with them regarding how to deal with people’s concerns and problems”.*

#### Learning or development of skills for helpers

All helpers rated the possibility to learn new skills and personal development by becoming PM + trainers or supervisors as positive. However, they clearly stated that they would need further training to do so. Helper 4 explained that they would *“need to learn management skills, how to facilitate dialogue, trainings, or workshops, in addition to time management and planning skills. All these are skills that supervisors need. It’s therefore important to have a training for those of us who will become supervisors, as far as I’m concerned. We also need some practice rounds”.*

Additionally, helper 3 said that if they were to become supervisors, they “*will continue to need their own supervisors. […] they will be supervisors, but they’ll continue needing that main supervision”*.

Furthermore, one helper (1) stated that offering helpers to develop and adopt a new role (e.g., becoming a supervisor), would bring on a new perspective, which is something that the helpers *“need for their life in Switzerland”*. One policy maker (1) pointed out that working with lay people from the same communities bears the risk that they are exploited by the deficient system and *“that [the helpers] are exploited to fill these deficiencies”*. Thus, according to this policy maker, providing helpers with a long-term perspective is crucial.

#### Supervision and support by organizing institution

While helpers said that they learned a great deal from supervision, they indicated there were some aspects which could be improved. Several helpers brought up that they didn’t feel comfortable discussing difficulties or sensitive matters in a group supervision and that having more opportunities for individual supervision sessions would have been ideal *(“It would be great to have the possibility to request a one-on-one with the supervisor and privately discuss a specific problem, or if one gets affected by a certain event”*, Helper 3; “*I would’ve loved to have individual supervision sessions. Because there were things I’d rather not speak of in a group, not too appropriate to be shared in a group setting”*, Helper 4*).*

Other suggestions for supervision improvement were greater efficiency by preparing tasks beforehand and sending them to the supervisor, as suggested by Helper 5, more interactivity during the sessions), and a greater variety of supervisors to reach out to, as suggested by Helper 4.

Healthcare providers and policy makers stressed the need for a permanent [emergency] support next to regular supervision. Healthcare provider 6 elaborated *“that they can always call, in case of a problem. That they always have a “back-up”, a permanent support system”.*

### Modality (PM + format)

Advantages and disadvantages of the different PM + formats (i.e., individual, group, and online PM+) are summarized in the following subthemes. It is important to note that while most interviewees mentioned advantages and disadvantages for each format, some interviewees also stated that for scaling-up the intervention, more than one format should be available for the beneficiaries to choose from. One healthcare provider (6) said: *“I almost think that you cannot avoid either offering all three [formats] or at least individual and group [PM+]”*. Similarly, healthcare provider 2 said: *“I think the most important thing would be that [the choice of format] can be adapted to the needs and that it’s not either just group or just individual [PM+]”.*

#### Group PM+

Helpers, healthcare providers and policy makers stated that a positive effect of group PM + could be that participants realizing that others suffer from similar problems and thus, they would feel less alone. One helper (4) said that group PM + also provides the *“opportunity to learn from others’ experiences, from the exchange of experiences and stories”*. However, interviewees from all KI groups stated that a group format makes it difficult to share personal issues. One PM + participant (2) illustrated this point by saying: *“I don’t think group sessions would work. Share our everyday personal issues and challenges during the sessions in the presence of others can be embarrassing and so some won’t feel comfortable sharing. I personally don’t like talking about my struggle in front of people”*.

#### Individual PM+

Interviewees from all KI groups agreed that individual PM + allows for greater confidentiality and encourages participants to open and share personal topics with their helper.

Furthermore, it was raised that a one-on-one setting would make it easier to establish a relationship between the helper and the participant. One helper (3) stated: *“I don’t remember anyone talking about issues affecting them personally, or about problems with their spouses or kids, in front of others. Never. Because trust is between two people. They know they can trust the helper”.*

#### Online PM+

Interviewees stated that online interventions are practical as participants and helpers do not need to commute (*“The advantages of online are the typical advantages. It is convenient, money-wise and distance-wise”*, policy maker 5). Hosting sessions online would also allow for participants from areas further away from implementation centers to attend. Additionally, healthcare provider 5 mentioned that for younger individuals, use of the internet and online tools is very common suggesting that they would be more likely to accept online interventions. One PM + participant (2) said that *“due to COVID, I started doing everything digitally. I got used to it so I had no issue doing it online”*.

Other interviewees expressed skepticism for online interventions, indicating that they could be perceived as boring or as one helper (4) said that “*a portion of online sessions is wasted on trying to fix sound and technical issues, which disturbs the flow and is more impersonal”*. Other negative aspects raised were the difficulty of opening up *(“I disliked online sessions because I feel more present and open during in-person sessions”*, PM + Participant 3), lack of privacy at the refugees’ homes (“*People come and talk about everything, including private things. This will not work if the children, husband or wife are in the room on the other side*”, Helper 1) and unstable internet connections may prevent individuals from participating.

### Views on task sharing

#### Compatibility of helpers and participants

Helpers and participants stated that from their experience, the compatibility between helpers and participants was mostly good. They stressed many benefits of engaging peers with the same cultural background: helpers went through similar experiences,, no language barrier, good understanding of the participants’ culture enabling trust and honesty. One helper (4) mentioned that while she was expecting that there would be gender issues, she experienced the opposite: *“There were some challenges I was expecting but didn’t experience, regarding a man learning from a woman. Usually in Middle Eastern societies, men are too proud to learn from women, so I was expecting that to happen and had asked to work with women only if possible. I expected men to totally reject working with me as a helper. But my experience proved the opposite. I’ve worked with an old man, then a young man, and in both cases, they were receptive, cordial, and great listeners. This was a great experience for me”.*

Still, a PM + participant (2) mentioned that gender concordant sessions would be good because *“there could be answers that a woman would be embarrassed to share with a man”.* Similarly, policy makers and healthcare providers mentioned that gender matching needs to be considered when pairing a helper and a participant, especially for victims of sexual abuse. Furthermore, healthcare provider 2 mentioned that social stratification is much stronger in other cultures and that *“it doesn’t necessarily mean that just because they come from the same culture, they also come from the same social class. […] [ it could be] that someone says, I won’t go to a group with someone who I don’t know what function he had in my country and talk about my problems there”*. In addition, some policy makers and healthcare providers pointed out that working with peers does not always promote trust and honesty as some refugees might mistrust individuals from their own country.

#### Competence of helpers

Regarding function and competence of helpers, interviewed helpers themselves stated that there needs to be a clear distinction between the role of a “helper” and the role of a licensed psychotherapist or psychiatrist. While helpers can deliver stress and problem management to individuals who have faced adversities and suffer from mild to moderate distress, it is not intended that they work with individuals suffering from severe mental health problems. Helper 1 illustrated this by saying *“I want to emphasize that again and again: We are neither psychologists nor therapists. We have just attended a training at the university. We are not trained for such difficult cases. Perhaps we need to define this better.”*

One healthcare provider (2) stressed that refugees often report psychosocial problems which can be addressed by a therapist but not by a helper. Thus, healthcare provider 2 stated that it would be difficult or even unethical to let peers deal with these problems, as they do not have the authority nor competence to solve them: “*If you [employ peers], who then feel with these people because they have also had the same or similar experiences, but on the other hand there are no possibilities to find solutions, then that is actually a bit difficult in my opinion and ethically questionable to expose the helpers to these situations*”.

While some healthcare providers and policy makers mentioned that refugees might prefer to receive treatment by a professional therapist because of their competence, one PM + participant (4) stated that *“the most important thing is that they’re great listeners. It’s not a condition for them to be specialized therapists. You just need to be able to trust them and talk to them without fearing anything”*.

#### Mental health of helpers

This subtheme summarized statements about the mental health of helpers and how their mental health could be affected by conducting PM + sessions. Healthcare providers and policy makers stated that working with peers carries risk of re-traumatization and expressed their worry that it would be difficult for them to keep a professional distance from participants as they have dealt with similar hardships (*“[Vicarious traumatization means that] the helpers are also traumatized by what they hear. […] So traumatization by hearing such stories … and that you get the feeling that the world is so unfair*”, Healthcare provider 2). No helpers mentioned that delivering PM + had a negative impact on them. One helper (3) said that while “ *[I can] definitely can relate to their sadness and depressive episode, […] I overcame it so I’m able to help people going through it. I’m more resilient and less likely to be negatively affected by people’s stories. That’s an old chapter, a memory now, and it doesn’t bother me anymore”*. Moreover, this helper also stressed that *“these strategies are beneficial to us too in the first place. Then, as I worked with other people and started to see how it was helping them too, even if it only helped them overcome 5% or 10% [of their issues], I was glad to be of service to them”*.

### Time and setting when PM + is offered

Advantages and disadvantages of the different times and settings when PM + is offered (after settling in vs. shortly after arrival) are summarized in the following subthemes. While interviewees stated preferences regarding the time and setting of PM + delivery, some interviewees also stated that the beneficiaries should participate when it is the “right” time for them. One helper (5) illustrated this by saying: *“There are always challenges to overcome; we’re constantly in need of support to understand our problems and ways we can overcome them. So the answer to your question needs to stem from the participants themselves, for them to decide when they’re ready to voice their concerns and seek help”.*

#### Shortly after arrival

Many KIs stated that it is best to provide PM + as soon as possible after arrival in Switzerland. Helper 3 said: “*Newcomers [in the asylum centers], arriving only a few months ago, would benefit greatly from the program. It’d help them with general communal problems they face in the camp as well. It would also be some form of a healthy escape from the […] life [in the asylum centers]*”. Similarly, one PM + participant (4) said that there is no stability until you have found a home. Healthcare providers and policy makers agreed that the sooner psychosocial support is offered, the better, especially because this approach facilitates “*recognizing at an early stage if someone is doing very badly. That is not 100% guaranteed at the moment”*, as mentioned by policy maker 4.

Nevertheless, in this KI group, it was frequently mentioned that asylum centers resources are limited and refugees are often concerned with legal and practical issues. One healthcare provider (2) illustrated this by saying: *“When people arrive, right at the beginning, they are often still in an intermediate state where they are quite happy to have arrived somewhere, to have a roof over their heads and to be able to catch their breath […]. But they often want very specific information: How do I do this? What’s the next step? I don’t know if they are really open to managing their problems differently at that point. So I don’t know if this is really the right moment [to offer PM+]”*.

#### After settling in

Offering PM + after refugees have settled into communities in Switzerland was regarded as more feasible by some interviewees as this approach allows for more continuity and sustainability, i.e. participants do not have to move around, thus they can finish all five sessions or complete all session with the same helper.

PM + participant 2 stated that the right time to offer PM + is after settling in as *“they gain a certain degree of independence, […] they have so much free time and so many old issues that come back to haunt them.”.* However, one former PM + participant (3) warned that waiting too long to offer psychosocial support could lead to a chronification of symptoms: *“If I had known about [PM+] beforehand, I wouldn’t have come to where I am now. The first 3 years I was stuck in one place”.*

## Theme 3: benefits of scaling-up

### Facilitated access to healthcare and reduction of care gap

One perceived benefit of scaling-up was that PM + could promote facilitated access to healthcare and reduce the treatment gap. One policy maker (2) said that a low intensity approach provided by helpers might be perceived as less stigmatizing compared to standard mental healthcare services provided by psychiatrists or psychotherapists as *“with refugees, psychological disorders are often very stigmatized, and seek for help. This [low-intensity] approach can […] be good, because you don’t see a doctor, but a peer”*. Similarly, healthcare provider 5 mentioned that while in Western contexts, we have our understanding of mental illnesses and treatment approaches, these concepts are *“not common for other kind of cultures and other societies. […] PM + is more adapted to some cultural expectations”.* Healthcare providers and participants agreed that individuals affected by adversities who recently settled in a new context need to be understood and heard. According to one healthcare provider (5), *“many times [refugees and asylum seekers] do not want to talk about [the traumatic experiences] and it is more about how to manage the symptoms and what happens now”.* One PM + participant (5) stated that PM + might facilitate access to care as it overcomes the language barrier and therefore makes it easier but also safer to access services: *“[When I came to Switzerland] I was feeling overwhelmed, family problems and so on. It was all weighing on me. I didn’t have my own space in the midst of a noisy communal life. The camp officers told me to sort it out on my own, but I didn’t speak the language at all, not even the basics. I needed to rely on my own, and I even had to attend appointments on my own. They’d tell me to go on my own. I’m headed to a doctor without knowing the language, miscommunicating could cause harm to myself”.*

Another crucial point mentioned by healthcare providers and policy makers was that implementation of a stepped care approach in Switzerland would relieve burden on institutions that specialize in providing trauma-focused psychotherapy. One policy maker (4) said: *“Especially the ones working in in-patient settings would be glad because they might have some relief, if [treatment is provided] at an early stage. […] in outpatient settings [they] are probably also grateful because they are often overwhelmed with the clients”*.

One healthcare provider (4) stated that the current situation in Switzerland with long waiting times, gatekeeping though GP’s (“Hausarztmodell”), worsening of symptoms and (involuntary) hospitalizations create a vicious cycle that is equally frustrating for refugees and asylum seekers and for professionals working with them.

### Supplementary offer

One policy maker stated that implementing low-intensity approaches would provide more possibilities to get help – as not all refugees need specialized treatments provided by highly qualified service providers. One policy maker (3) illustrated this by saying: *“You realize that a highly specialized offer is not right for everyone either. That there needs to be a greater range to get help”.* In the same vein, one healthcare provider (6) said that to provide everyone with adequate treatment, it needs more than just standard mental health care: *“It needs supplementary offers. We need to offer different services to fulfill everyone’s needs”*.

### Support during asylum and integration process

Helpers and participants similarly stated that PM + not only reduces mental distress, but is also a step towards social integration, as one helper (2) said: *“One can’t integrate without the ability to functionally address and tackle one’s problems”*. One PM + participant (2) said that meeting fellow Syrians and exchanging views on the integration process was one benefit of the PM + intervention:

*“I rarely get together with Syrians. We are looking to integrate within the society here but it is still great to surround oneself with a community who shares similar experiences and has a deeper knowledge of one’s life circumstances, and therefore able to exchange views on it. That, too, was a great aspect about this program”*.

## Discussion

The aim of the present study was to identify factors influencing the process of the large-scale implementation of PM + for refugees in Switzerland and to develop recommendations to guide the implementation process. The study revealed three major themes which may have implicatons for longer-term implementation of PM + in Switzerland. The first theme consisted of requirements for successful integration in the health care system prior to scaling-up, while the second theme related to requirements for the PM + intervention supporting scale-up such as the modality of the intervention. The last theme highlighted the perceived benefits of scaling-up PM + for refugees in Switzerland.

In the first theme of “Requirements for successful integration in the health system,” one prominent issue was to secure sustainable funding for the implementation. Funding by a private institution was not perceived as sustainable, and interviewees stated that the implementation should be funded by a governmental insitutions to be sustainable. Depending on the aim of scaling-up PM+ (prevent symptoms of mental disorders vs. teaching strategies how to deal with psychological impairment) different sectors could be potential funders of the intervention. Policy makers stated that ideally, PM + sessions could be funded by the public health insurances. As it might take time and effort to to meet the high requirements and go through the complex processes necessary for the recognition of a low-intensity psychological intervention delivered by non-specialists” as an insurable service, a combined approach – initial funding by NGO’s or the private sector while initiating the recognition process of the intervention with health insurance companies – seemed most promising. Similarily, Yamey [56] stated that it is beneficial to cooperate with both governmental and non-governmental providers to support scaling-up heath intervetions as this approach combines stability and long-term perspective of govermental funding with the agility and flexibility of non-governemental institutions.

Another requirement for successful integration in the health system was to ensure a stepped-care approach, including a functioning triage system. As many interviewees argued, one potential difficulty might be that (immediate) care in specialized treatment facilities needs to be accessible in case someone is too distressed to participate in PM + or if, someone still needs additional treatment after completing PM+. Currently, there is a lack of capacity of specialized treatment for refugees in Switzerland, resulting in long waiting times (more than one year for a first consultation) [25, 26, 57, 58]. While these long waiting times are a structural problem which might be tackled by the introduction of stepped-care approaches, a thorough triage process with clearly defined levels of the stepped care model is crucial to avoid “misplacement” of individuals. One example of the introduction of a stepped care approach in a high-income country is the MEHIRA study that was carried out in several cities in Germany [59]. While the results on effectiveness and cost-effectiveness are promising, the authors highlighted the organizational complexity of implementing and delivering a stepped-care model [59].

In the second theme of “Requirements for PM + intervention supporting scale-up” one major requirement was quality control during the delivery of PM+, during the selection process, the training of new helpers and the supervision of helpers. Helpers highlighted that while selection criteria such as good knowledge of the local language, a potential helper also needs soft skills, such as active listenting skills and empathy. While hard skills are easily assessible in the CV, basic helping skills (e.g., validating or putting aside your personal values) [60] and PM + competence could be assessed using role plays and the EQUIP rating tool after the training and during PM + delivery [61–64]. Additionally, one helper suggested more experienced helpers could assist the implementing organizations in the selection process and the training of future helpers. Engaging experienced helpers in selection, training, and supervision of new helpers was healthcare providers and policy makers also endorsed as it offers the more experienced helpers a possibility to develop long-term perspectives and ultimaltely also benefits scaling-up.

Another important aspect of the theme “quality control” was supervision. While supervision was regarded as helpful, some points for improvement were mentioned (e.g., have both group and individual supervision). Chiumento and colleagues [65], who delivered and implemented PM + in the UK, offered both individual and group supervision. Individual supervision took place immediately after PM + sessions, as supervisors were present during the delivery of PM+. Having supervisors present during PM + sessions and offering immediate individual supervision if needed would be one method to offer further support. A way to save resources but still provide both supervision formats could be to have regular group supervision led by experienced PM + helpers and an individual supervision, ideally directly after the PM + delivery, by specialized mental health professionals. Offering peer-supervision might increase acceptability of supervision sessions, as Singla and colleagues have found that trained lay-therapists prefer supervision delivered by peers compared to supervision led by mental health specialists [66].

Another requirement regarding PM + concerned modality of implementation. In general, individual PM + seemed to be the preferred modality among all interviewees. Online delivery was frequently described as a compromise, as it saves time and increases the reach of the intervention, but risks lack of privacy in asylum shelters, and online devices are needed to receive the intervention. While helpers, healthcare providers and policy makers highlighted some advantages of group PM+ (e.g., social support, learning from other group members), none of the former PM + participants could imagine participating in a group format.

However, rather than selecting one modality for implementation, it seemed preferable to offer a variety of formats and let beneficiaries decide their preference. A meta-analysis on the impact of accomodating client preference in psychotherapy has shown that clients who receive their preferred treatment options show higher improvement and lower rates of drop out [67]. There would likely be a similar effect in low-intensity interventions. Thus offering different modalities and letting beneficiaries choose seems the most acceptable and effective approach. Nonetheless, the cost-effectiveness of the different modalities would need to be examined. In future research, it could be interesting to focus on which modality works for whom and to delevop guidelines to achieve a maximum fit between the beneficiaries and the offered PM + modalities.

A similar approach can be drawn when determining a time point or setting for PM + delivery. One helper stated that the time and setting for PM + delivery can only be defined by beneficiaries becauses it requires them to be ready for such an intervention. In theory, PM + could be delivered in federal asylum centres (i.e., shortly after arrival) or under cantonal structures, once refugees have settled. Interviewees from all KI groups advocated to intervene and provide mental health support as soon as possible to prevent clinical detoriation. This statement resonates with findings from multiple studies that the length of stay in the host countries, especially a prolonged stay in asylum shelters and a lengthy asylum procedure, has a detrimental effect on the mental health of refugees [27, 68, 69]. However, one interviewee also raised concerns whether beneficiaries would be able to follow and benefit from such an intervention at an early stage as they are frequently challenged with pressing legal and pratical issues. While at the beginning, many of the beneficiaries might have practicial questions (e.g., about their asylum claim), after they have received a decision on their residence status, they are confronted with other stressors such as social and economic integration [8, 11, 12].

When talking about competence of helpers, one healthcare provider was concerned that beneficiares would have psychosocial problems that cannot be addressed by the PM + intervention. While refugees are confronted with a great variety of psychosocial problems, literature on how to successfully integrate these psychosocial problems into treatment approaches are scarce [70, 71]. A recent study by Spaaij and colleagues [45] shows that refugees who participated in PM + report significantly fewer post-migration living difficulties compared to refugees who have received treatment as usual. These results suggest that PM + has the potential to effectively deal problems faced by refugees. Nonetheless, adapting PM + to the setting and the population, and developing additional PM + modules (e.g., a module regarding alcohol abuse or emotional processing) seems promising to further address beneficiaries needs and is something that is currently underway [72–74].

The views on task sharing differed significantly between the different KI groups. While the policy makers and healthcare providers anticipated difficulties regarding the competence of helpers and the compatability with participants, and anticipated that delivering PM + might have negative impacts on the mental health of the helpers, helpers and participants felt differently. One such example was the perceived risk of re-traumatization or vicarious traumatization which was raised by some healthcare providers and policy makers. Nonetheless, none of the helpers stated that delivering PM + has harmed them in any way. In contrast, one helper described that being a PM + helper had been a positive experience and that learning PM + strategies had personally benefited them. A review by Shamalak and colleagues [75] on the experiences of non-specialized workers delivering psychosocial interventions has found a similar effect, with most stating that they personally benefited from their work by delevoping new skills and improving their own interpersonal relationships. While the mental health of the helpers is extremely important to consider and protect, it is equally important to emphasize the positive effects of PM + on their wellbeing. Regardless, self-care and mental hygiene should be an integral part of the training of helpers as well as supervision to prevent mental health distress.

The third theme on the “Benefits of scaling up” related to potential outcomes of scaling-up PM + in Switzerland. One benefit of scaling-up PM + in Switzerland was facilitated access to health care and a reduction of the current care gap for refugees in Switzerland. A nationwide implementation of PM + facilitates access to healthcare on a structural level, including the possibility of relieving trauma focused care workers, reduce waiting times for beneficiaries and partly by-passing language barriers and lack of resources for interpreters.

Low-intensity, psychosocial programs might also reduce stigma surrounding mental health and may be more tailored to what refugees may require [76]. Thereby, implementing PM + may help counteract structural and socio-cultural barriers that limit possibilities and desires for refugees to seek healthcare, as described by Kiselev and colleagues [26].

Scaling up PM + in Switzerland may also diversify the range of care options available for refugees. In KI interviews, PM + participants mentioned that it does not matter if the person they spoke to was a specialist or a peer, they just needed someone to listen to them. Moreover, many of the problems refugees experience are not necessarily of a psychiatric nature but may be psychosocial due to the difficult circumstances they live in [21, 22]. Savic and colleagues [77] discussed that refugees might not share Western mental health beliefs and thus, may not want treatment by healthcare providers or psychiatrist. Their road to recovery might not follow a medical approach, but a social one, e.g., by receiving support from their community [77–79].

## Limitations

As some interviewees (former PM + participants and PM + helpers) were recruited from a larger project testing the feasibility and (cost-) effectiveness of PM + among Syrian refugees, the population was limited to the views of one ethnicity. In recent years, there has been an influx of refugees coming from Eritrea, Afghanistan, Turkey [23] and most recently, Ukraine [24]. The country of origin may have an influence on the daily life of refugees and their post-migration stressors, including their ability to stay in Switzerland, work, integration in the community. To fully represent healthcare barriers and perspectives on whether scalable interventions would be appropriate in these populations, future initiatives should include representation from different refugee groups.

Another limitation of this study was the sampling method. All interviewees knew one of the research team members as they participated in the interviews upon invitation by the research team. Using a different sampling method such as snowball sampling, would have allowed for a greater variety in perspectives related to this study and might reduce sampling bias. Furthermore, while our overall sample consisted of 22 interview participants, there were only five participants for most subgroups. Thus, it is likely that data saturation had not been reached for individual key informant groups, as they might not be very homogeneous groups [80]. In future research, it would be worth including more participants per group [80] or adopting a study design where researchers actively check for data saturation during data collection.

Lastly, the PM + participant group was composed of individuals who had previously received and attended PM+. In future research, it would be beneficial to conduct in-depth qualitative interviews with individuals who have not yet participated in PM + or who had previously dropped out.

## Implications and conclusion

Our findings have implications for future research, as well as the scale-up of low-intensity interventions, such as PM+. It became evident that there is no ideal PM + modality or setting but that it seemed important to offer a variety of PM + formats to achieve maximum reach and benefits. In future research, it could be interesting to get a deeper understanding of what works for whom and to create guidelines which could be used in the process of scaling-up. Moreover, it was mentioned that refugees face different problems depending on where they stand in the asylum procedure. Shortly after arrival, many of them are concerned with legal and practical issues, while later they face issues regarding social and economic integration. It could be promising to adapt the generic version of PM + to the temporal needs of the beneficiaries and develop additional PM + modules (e.g., a module focusing on typical post-migration living difficulties in Switzerland). Regarding quality control during PM + delivery, helpers mentioned suggestions for improvement, concerning the selection process, the training of helpers and the supervision, which can be used for scale-up. The views on task sharing differed between helpers and participants, who had hands-on experience with PM+, and healthcare providers and policy makers, who have not worked with the intervention previously. The contradictory views (e.g., on gender concordance, on the competence of helpers or on the mental health of helpers and the risk of re-traumatization) show the need for awareness campaigns on low-intensity interventions not only for beneficiaries, but also for specialized staff working with refugees. To achieve successful scale-up of PM + in Switzerland, the intervention does not only need to be adopted by the target group, but also by already existing services. Our results have shown that a successful scale-up of PM + in Switzerland might have various benefits such as facilitated access to healthcare and a reduction of the mental health care gap, but also that PM + would diversify the range of care options available for refugees and provide the beneficiaries with support during their asylum and integration process. While these potential benefits need to be monitored throughout scale-up initiatives, communicating them to policy-makers and health providers might enhance their acceptability of the intervention and willingness to adopt PM + in regulatory structure and promote it. Communicating these benefits could also increase the likelihood that policy makers will allocate funds for implementation; especially if future research confirms that PM + is not only effective reducing symptoms of mental disorders, but also cost-effective compared to current treatment options.

## Electronic supplementary material

Below is the link to the electronic supplementary material.

Supplementary Material 1

Supplementary Material 2

## Data Availability

The data generated and analyzed during the current study are not publicly available due the qualitative nature of the data and the need to ensure the anonymity and confidentiality of the participants. The data can be made available from the corresponding author on reasonable request.

## List of Abbreviations

UNHCR: United Nations High Commissioner for Refugees
PM+: Problem Management Plus
KI: Key informants
