# Supplementary material for: Scaling-up problem management plus for refugees in Switzerland - a qualitative study
Source: BMC Health Serv Res. 2023 May 15;23:488. doi: 10.1186/s12913-023-09491-8 (PMC10186708; doi:10.1186/s12913-023-09491-8)
Supplement: Supplementary file 1 — Supplementary Material 1 [file 12913_2023_9491_MOESM1_ESM.docx]

# Key Informant Topic Guides

# Helpers

# PART 1: Introduction

Thank you for agreeing to participate in this interview. I would like to briefly explain the purpose of this interview. Refugees often suffer from stress and mental strain. However, proper treatment for these problems is far too rare in Switzerland. For this reason, the STRENGTHS research project has investigated the use of PM+ with refugees. Our research group is now working on a new project called "SPIRIT", which aims to make PM+ available to all refugees. To ensure this is successful, we have come up with three building blocks: In addition to PM+, we also want to introduce an awareness campaign and screenings to assess mental stress. As we are still at the beginning of the project, we are currently planning a strategy on how to offer these three building blocks to everyone in a sustainable and most effective way. The information from this interview will be used for these considerations. The interview will consist of three sections, PM+, screenings, and awareness campaign, and will last approximately one hour. In addition, the interview will be recorded. The recording will now be started. Do you agree to this?

I would like to remind you that you can skip individual questions or withdraw from the entire interview at any time. The information you have provided up to the point of withdrawal will be used for the evaluation. Did you have time to read the study information? Did you understand everything or did any questions arise for you? Ms/Mr XY, can you confirm verbally that you have read, understood and agree to the study information and the consent form attached at the back?

**Question regarding PM+**

| Questions | Probes | Notes (note gender) |
| --- | --- | --- |
| Now, could you please describe your age, position in the context of PM+ |  |  |

| Fragen | Probes | Notes |
| --- | --- | --- |
| 1. Could you briefly explain the most important things you have learned from the delivery of PM+? | *-advantages/disadvantages for the service user and provider*  *- structural challenges/barriers*  *- specific feedback from the participants* |  |
| 1. What have been some of the benefits and challenges of providing PM+? | *- helping others/community; - building own capacity/training*  *- responsibility*  *- time burden*  *- incentive/motivation*  *- opportunity costs*  *- psychosocial burden*  *- quality control*  *- resources required to effectively deliver* PM+ |  |
| 1. What do you think are the main reasons why some refugees would choose not to participate in a programme like this? | *- time commitment*  *- stigma about seeking help for mental health problems*  *- acceptability of features of the helper (e.g. non-professional)* |  |
| 1. During your Helper activity, you regularly participated in supervision sessions. What do you think about how the supervision was conducted? What could be improved about the supervision? | *- frequency*  *- utility*  *- confidentiality* |  |
| 1. During the PM+ intervention you worked in the field of psychosocial care, although your professional background is a different one. What problems and barriers did you experience? | *- responsibility*  *- opportunities for training*  *- support*  *- supervision*  *- understanding of one’s role as a helper* |  |
| 1. Ideally, after some time, experienced helpers could become supervisors and PM+ trainers. What is your view on this process? What support would you need to take on the next step? |  |  |
| 1. In your view, do you think there is a need for an intervention like PM+ to be scaled up in Switzerland? | *- advantages over existing mental health services*  *- advantages for the health system as a whole (strengthening of health system)*  *- advantages for target population*  *- long-term vision for PM+? (e.g. the societal problem addressed by PM; what ‘transition’ might scaling-up PM+ contribute to)* |  |

| 1. What do you think are the main obstacles for scaling up PM+ in Switzerland? Thank you for explaining some of the obstacles. I am interested to hear your thoughts on what is needed to overcome these obstacles ; could you tell me more about this? | *Structure:*  *- official recognition of their work (e.g., in terms of career development).*  *- physical access to PM+ for all*  *Practise:*  *- quality control*  *- prevention from becoming an emotional burden for Helpers*  *Culture:*  *- stigmas*  *- help-seeking*   - *Helpers being accepted by participants and other health workers?* |  |
| --- | --- | --- |
| 1. PM+ can be offered in different formats: individual session, in a group format or online. How was your experience and what preferences do you have for the delivery mode (online, individual, group) of the intervention? What could be potential advantages and disadvantages of each format? |  |  |
| 1. In your opinion, at what time point e.g. shortly after arriving in Switzerland or after resettling in the communities, does it make sense to take part in PM+? From your experience, at what time point can participants benefit the most? |  |  |
| 1. PM+ is based on ‘task-shifting’, meaning shifting tasks from professionals to non-health professionals. What are in your view the challenges of ‘task-shifting’ services like PM+ for refugees in your country? How can these challenges you mentioned be overcome when PM+ will be scaled up? | - *acceptability* - *training* - *supervision* |  |
| 1. Are there other factors that you think might be hindering or helping with regard to PM+ implementation that have not yet been addressed? |  |  |

# Health Providers and Policy Makers

**PART 1: Introduction**

Thank you for agreeing to participate in this interview. This interview takes place within the framework of the research projects SPIRIT and STRENGTHS. The goal and task of both projects is to provide refugees in Switzerland with better access to treatment for psychological disorders and to strengthen their overall psychological resilience. It has been observed for years that refugees are increasingly suffering from disorders such as depression or post-traumatic stress disorder. We know from studies that refugees receive treatment for these mental illnesses much less frequently. STRENGTHS has investigated the use of the Problem Management Plus intervention with refugees in response to this shortcoming. Based on these studies, SPIRIT now wants to offer the intervention PM+ throughout Switzerland in order to change this gap in care. In addition to PM+, two other components are to be developed and offered: an awareness campaign and screenings to assess mental stress. The Spirit project is still at a very early stage of funding and the current focus is on the strategic planning of how we can use these three elements to initiate a sustainable change in the Swiss system. Using the information from the interview, we would like to develop a strategy on how to successfully implement PM+, the screenings and awareness campaign. The interview will last approximately one hour and will be recorded. The recording will now be started. Do you agree to this?

I would like to remind you that you can skip individual questions or withdraw from the entire interview at any time. The information you have provided up to the point of withdrawal will be used for the evaluation. Did you have time to read the study information? Did you understand everything or did any questions arise for you? Ms/Mr XY, can you confirm verbally that you have read, understood and agree to the study information and the consent form attached at the back?

| Question | Probes | *Note: Gender* |
| --- | --- | --- |
| 1. Now, could you please describe your age, position and experience with PM+ | *explain PM+ if necessary* |  |

| Question | Probes |  |
| --- | --- | --- |
| 1. In your opinion, is there a need to implement an intervention like PM+ in Switzerland and offer it nationwide? Please explain. | *Advantages over existing mental health services*  *Benefits to the health care system as a whole (strengthening the health care system).*  *Long-term vision for PM+? (e.g., the societal problem addressed by PM; what "transition/change" might an expansion of PM+ contribute to)* |  |
| 1. What do you think are the obstacles if we now want to offer PM+ not only as a single project in Zurich, but make it available to all refugees in Switzerland? Thank you for explaining some of the obstacles. I am interested to hear what is needed from your perspective to overcome these obstacles. Could you tell me more about that? | *Structure:*  *Physical access for participants*  *Policies*  *Political will*  *Funding*  *Practise:*  *(emotional/time) burden on helpers and supervisors?*  *Culture:*  *Stigma*  *help seeking*  *acceptance of helpers by other progessionals* |  |
| 1. What could be possible supporting institutions, or from which sector should these institutions come? | *Advantages and disadvantages of the sector* | (adapt this question to the person) |
| 1. What do you think are the reasons why some refugees would not participate in such a program? |  |  |
| 1. In your opinion, at what time point of the migration process, e.g. shortly after arriving in Switzerland or after moving in cantonal localities, does it make sense to take part in PM+? At what time point can participants benefit the most? Please explain why. |  |  |
| 1. PM+ can be offered in different ways: individually, in a group format or online. What preferences do you have for the delivery mode (online, individual, group) of the intervention? What could be potential advantages and disadvantages of each format? |  |  |
| 1. PM+ is based on ‘task-shifting’, meaning shifting tasks from professionals to non-health professionals. What are in your view the challenges of ‘task-shifting’ services like PM+ for refugees in Switzerland? How can these challenges you mentioned be overcome when PM+ will be scaled up? | *Acceptability for potential organisations* |  |
| 1. How do you think quality control and accountability can best be ensured when scaling-up PM+? | *spontaneous scaling-up/mushrooming*  *sustainability*  *training* |  |
| 1. Are there other factors that you think might be hindering or helping with regard to PM+ implementation that have not yet been addressed? |  |  |

**PM+ participants**

**PART 1: Introduction**

Thank you for agreeing to participate in this interview. I would like to briefly explain the purpose of this interview. Refugees often suffer from stress and mental strain. However, proper treatment for these problems is far too rare in Switzerland. For this reason, the STRENGTHS research project has investigated the use of PM+ with refugees. Our research group is now working on a new project called "SPIRIT", which aims to make PM+ available to all refugees. To ensure this is successful, we have come up with three building blocks: In addition to PM+, we also want to introduce an awareness campaign and screenings to assess mental stress. As we are still at the beginning of the project, we are currently planning a strategy on how to offer these three building blocks to everyone in a sustainable and most effective way. The information from this interview will be used for these considerations. The interview will consist of three sections, PM+, screenings, and awareness campaign, and will last approximately one hour. In addition, the interview will be recorded. The recording will now be started. Do you agree to this?

I would like to remind you that you can skip individual questions or withdraw from the entire interview at any time. The information you have provided up to the point of withdrawal will be used for the evaluation. Did you have time to read the study information? Did you understand everything or did any questions arise for you? Ms/Mr XY, can you confirm verbally that you have read, understood and agree to the study information and the consent form attached at the back?

| Question | Probes | *Note: Gender* |
| --- | --- | --- |
| Could you first tell me a little bit about yourself?   - *age,* - *where currently reside,* - *how long been living in Switzerland,* - *whether in Switzerland with family and/or awaiting family reunification,* |  |  |
| If you are comfortable, could you briefly tell me for which problem you sought help via PM+. *Probe:*   - *type of problem,* - *when it started* |  |  |

| 1. How have you found using PM+? What did you like about it? What didn’t you like? What do you think could be improved? | *Structure*  *- access to program(Travel, location Flexibility of appointments, amount of time spent on homework)*  *- Further use of learned skills*  *Practise:*  *- sufficient to deal with your problems or further support needed*  *Culture:*  *- acceptance of the helper (lay person, refugee, male/female)*  *- participation been discussed with family*  *- change in ideas about mental health as a result of participation* |  |
| --- | --- | --- |
| 1. The programme that you have participated in can be offered in different ways: individually, in a group format or online. How was your experience and what preferences do you have for the delivery mode (online, individual, group) of the intervention? What could be potential advantages and disadvantages of each format? |  |  |
| 1. In your opinion, at what time point, e.g. shortly after arriving in Switzerland or after moving in cantonal localities, does it make sense to take part in PM+? At what time point would you have benefited the most? Please explain why. |  |  |
| 1. What were your reasons for joining this programme? What do you think are the main reasons why some refugees would choose not to participate in a programme like this? | *- time commitment*  *- stigma about seeking help for mental health problems*  *- acceptability of features of the helper (e.g. non-professional)* |  |
| 1. In your view, do you think there is a need for an intervention like PM+ for refugees in Switzerland? Please explain. | *- advantages over existing mental health services*  *- groups who profit more / less from intervention* |  |
