# Supplementary material for: Scaling-up problem management plus for refugees in Switzerland - a qualitative study
Source: BMC Health Serv Res. 2023 May 15;23:488. doi: 10.1186/s12913-023-09491-8 (PMC10186708; doi:10.1186/s12913-023-09491-8)
Supplement: Supplementary file 2 — Supplementary Material 2 [file 12913_2023_9491_MOESM2_ESM.docx]

| **Theme 1: Requirements for successful integration in the health care system before scaling-up** | | |
| --- | --- | --- |
| ***Subtheme*** | ***Definition*** | ***Example*** |
| 1.1 **Ensure stepped care approach** | Contains statements regarding the importance of a stepped care approach for the successful implementation of PM+, including a function triage system and the possibility for follow-up care, if needed. | *“If a low-intensity intervention reveals that cases are more severe, then it must also be possible to [offer further treatment]. … It's all very well to say after 5 sessions that this person actually needs psychotherapy. But what do you do next? Because the translator costs are not yet paid everywhere in Switzerland and there are not enough treatment opportunities for these people with specialized psychotherapists who are familiar with these topics. So you ask yourself, what's the use of early diagnosis if you can't follow up on it? It's like when you say you're screening for cancer and then you say: you do have cancer, but we can't perform surgery.” (Healthcare provider 2)* |
| 1.2 **Sustainable funding** | Contains statements on the importance of sustainable funding and the advantages and disadvantages of various funding options. | *«... from a financing point of view, we would be glad if [the implementation] is close to the health system, because then [PM+]. can also be billed to the health insurance. This is the cost-conscious policy maker talking. But from the perspective of the people concerned, the financing doesn't matter. But if, of course, it can be [implemented]. in the regulartory structures, like in health insurance, then it is simply easier to launch and use such offers". (Policy maker 2)* |

**CODING FRAMEWORK: Scaling-Up Problem Management Plus for refugees in Switzerland - a qualitative study**

| **Theme 2: Requirements for PM+ intervention supporting scale-up** | | | |
| --- | --- | --- | --- |
| ***Subtheme*** | ***Code*** | ***Definition*** | ***Example*** |
| **2.1 Quality control during delivery of PM+** |  |  |  |
|  | 2.1.1. Characteristics of helpers in the selection process | Statements on what needs be considered when selecting new helpers. | *“… the selection criteria shouldn’t be limited to having a university diploma and having spent a few years as a refugee in Switzerland. There are additional criteria that need to be taken into consideration. […] Trustworthiness, for instance, as well as awareness and active listening skills, management skills, punctuality and readiness when submitting their paperwork, and so on. A certain foundation or basis.” (Helper 4)* |
|  | 2.1.2. Training of helpers | Statements on how the training of new helpers could be improved. | *“[…] you would help them by providing them with real-life situations/examples during their training. There are many cases, […] which we can share with them regarding how to deal with people’s concerns and problems. […]. We’ve learnt basic things with a few examples, but now there is abundance in case studies and similar experiences that happen time and again. Additionally, if possible, we, the more experienced helpers, would join the new ones in the training and further provide assistance with the experience” (Helper 2)* |
|  | 2.1.3. Learning or development of skills for helpers | Statements regarding what helpers would need to develop new skills (e.g., become co-trainers, co-supervisors, etc.) | *“[…] need to learn management skills, how to facilitate dialogue, trainings, or workshops, in addition to time management and planning skills. All these are skills that supervisors need. It’s therefore important to have a training for those of us who will become supervisors, as far as I’m concerned. We also need some practice rounds.” (Helper 4)* |
|  | 2.1.4. Supervision and support by organizing institution | Statements on how the supervision and the support of helpers could be improved. | *“I would’ve loved to have individual supervision sessions. Because there were things I’d rather not speak of in a group, not too appropriate to be shared in a group setting.” (Helper 4)* |
| **2.2 Modality (PM+ format)** |  |  |  |
|  | 2.2.1. Group PM+ | Statements regarding the advantages and disadvantages of group PM+. | *“I don’t think group sessions would work. Share our everyday personal issues and challenges during the sessions in the presence of others can be embarrassing and so some won’t feel comfortable sharing. I personally don’t like talking about my struggle in front of people.” (Participant 2)* |
|  | 2.2.2. Individual PM+ | Statements regarding the advantages and disadvantages of individual PM+. | *“In individual sessions, participants are able to discuss the personal issues they don’t want others to know about, issues that would be affecting their character, behavior, and family life.” (Helper 3)* |
|  | 2.2.3. Online PM+ | Statements regarding the advantages and disadvantages the online delivery of PM+. | *“There’s no commute and hence less wasted time. The time it usually takes to get there is the time it takes me to complete a session.” (Participant 6)* |
|  |  |  |  |
| **2.3. Views on task sharing** |  |  |  |
|  | Compatibility of helpers and participants | Statements regarding the compatibility of participants and helpers (e.g. ethnicity / cultural identity), educational background, gender) | *"Sometimes they might speak the same language but they are not of the same ethnicity, cue Kurds from Turkey, they speak Turkish but wouldn't for the world want to be in a group with Kurds to talk about being tortured by Turks somehow". (Healthcare provider 2)* |
|  | Competence of helpers | Statements regarding the role of helpers and their competences (e.g. statements saying that the helpers are not psychotherapists and do not have the same competences). | *“I want to emphasize that again and again: We are neither psychologists nor therapists. We have just attended a training at the university. We are not trained for such difficult cases. Perhaps we need to define this better.”(Helper 1)* |
|  | Mental health of helpers | Statements regarding the helpers’ mental health, e.g. regarding their vulnerability, the risk of re-traumatization or their resilience. | *“… [I can] definitely can relate to their sadness and depressive episode, […] I overcame it so I’m able to help people going through it. I’m more resilient and less likely to be negatively affected by people’s stories. That’s an old chapter, a memory now, and it doesn’t bother me anymore.” (Helper 3)* |
| **2.4. Time and setting when PM+ is offered** |  |  |  |
|  | Shortly after arrival | Statements regarding the advantages and disadvantages of offering PM+ shortly after arrival. | *“When people arrive, right at the beginning, they are often still in an intermediate state where they are quite happy to have arrived somewhere, to have a roof over their heads and to be able to catch their breath […]. But they often want very specific information: How do I do this? What's the next step? I don't know if they are really open to managing their problems differently at that point. So I don't know if this is really the right moment [to offer PM+].” (Healthcare provider 2).* |
|  | After settling in | Statements regarding the advantages and disadvantages of offering PM+ after participants have settled in and live, e.g., in community settings. | *“As soon as they gain a certain degree of independence, I believe that’s when it’s best to offer help. Because then they have so much free time and so many old issues that come back to haunt them.” (Participant 2)* |

| **Theme 3: Benefits of scaling-up** | | |
| --- | --- | --- |
| Subtheme | Definition | Example |
| 3.1. Facilitated access to healthcare | Scaling-up PM+ could improve access to healthcare for refugees (e.g. in terms of availability, no language barrier, less mental health stigma, greater reach). | *“[When I came to Switzerland] I was feeling overwhelmed, family problems and so on. It was all weighing on me. I didn’t have my own space in the midst of a noisy communal life. The camp officers told me to sort it out on my own, but I didn’t speak the language at all, not even the basics. I needed to rely on my own, and I even had to attend appointments on my own. They’d tell me to go on my own. I’m headed to a doctor without knowing the language, miscommunicating could cause harm to myself” (PM+ participant 5)* |
| 3.2. Supplementary offer | A successful scale-up of PM+ would allow for more flexible treatment options and thus increases the likelihood of finding a suitable treatment option. | : *“…it needs supplementary offers. We need to offer different services to fulfill everyone’s needs” (Healthcare provider 6)* |
| 3.3. Support during asylum and integration process | Scaling-up PM+ would provide refugees additional support during their asylum and integration process. | “*PM+ [Refugees] is a step towards integration. [Refugees] would settle psychologically and spiritually, they’d calm down and loosen up, which is essential to successful integration. One can’t integrate without the ability to functionally address and tackle one’s problems.*” (Helper 2) |
